# Crystal structure of (*Z*)-1-(3,4-dichlorophenyl)-3-methyl-4-[(naphthalen-1-yl­amino)(*p*-tolyl)methylidene]-1*H*-pyrazol-5(4*H*)-one

**DOI:** 10.1107/S1600536814017140

**Published:** 2014-08-01

**Authors:** Naresh Sharma, Sanjay Parihar, R. N. Jadeja, Rajni Kant, Vivek K. Gupta

**Affiliations:** a Post-Graduate Department of Physics & Electronics, University of Jammu, Jammu Tawi 180 006, India; b Department of Chemistry, Faculty of Science, The M.S. University of Baroda, Vadodara 390 002, India

**Keywords:** crystal structure, Schiff base, naphthalene, pyrazolone, pyrrole

## Abstract

The title Schiff base compound, C_28_H_21_Cl_2_N_3_O, was synthesized by the condensation of 1-(3,4-di­chloro­phen­yl)-3-methyl-4-(4-methyl­benzo­yl)-1*H*-pyrazol-5(4*H*)-one with 1-aminona­phthalene. The *p*-tolyl ring is normal to the pyrazole ring, with a dihedral angle of 88.02 (14)°, and inclined to the naphthalene ring system by 78.60 (12)°. The pyrazole ring is inclined to the naphthalene ring system and the di­chloro-substituted benzene ring by 63.30 (12) and 11.03 (13)°, respectively. The amino group and carbonyl oxygen atom are involved in an intra­molecular N—H⋯O hydrogen bond enclosing an *S*(6) ring motif. There is also a short C—H⋯O contact involving the carbonyl O atom and the adjacent benzene ring. In the crystal, mol­ecules are linked by C—H⋯π inter­actions, forming a three-dimensional structure.

## Related literature   

For the preparation and biological activity of pyrazolo­nes and their metal complexes, see: Chiba *et al.* (1998 ▶); Xu *et al.* (2000 ▶); Casas *et al.* (2007 ▶); Wang *et al.* (2007 ▶). For Schiff bases and their diverse biological activity and exceptional chelating ability, see: Karthikeyan *et al.* (2006 ▶); Sinha *et al.* (2008 ▶); Jadeja *et al.* (2012*a* ▶,*b* ▶). For related structures, see: Sharma *et al.* (2012 ▶); Abdel-Aziz *et al.* (2012 ▶).
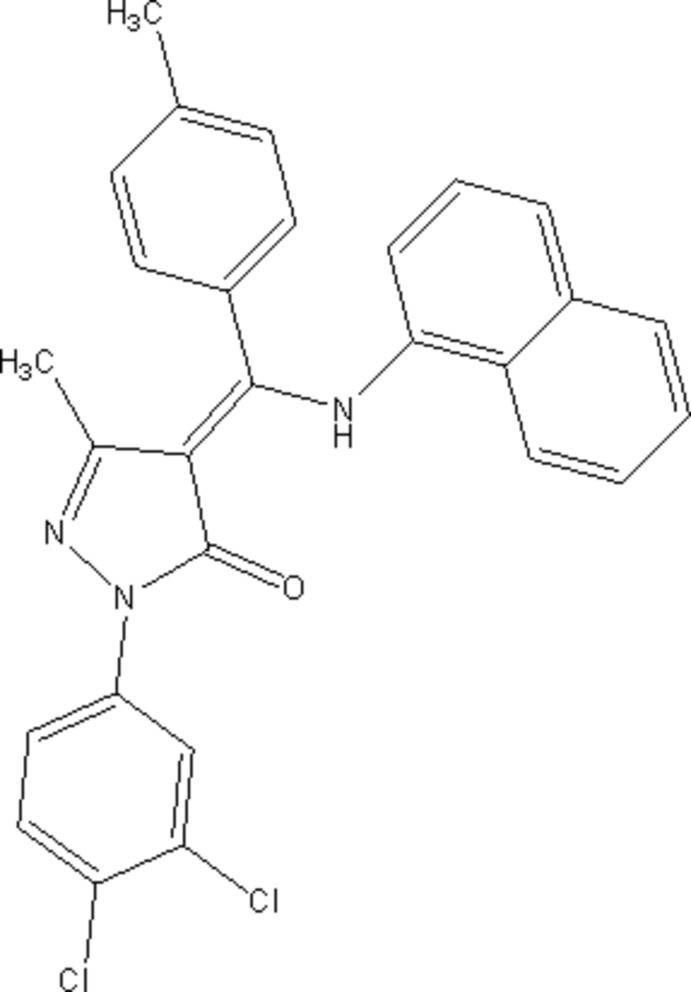


## Experimental   

### Crystal data  

C_28_H_21_Cl_2_N_3_O
*M* *_r_* = 486.38
Monoclinic, 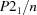

*a* = 11.4621 (8) Å
*b* = 16.9351 (11) Å
*c* = 12.2704 (9) Å
β = 97.478 (6)°
*V* = 2361.6 (3) Å^3^
*Z* = 4
Mo *K*α radiation
μ = 0.30 mm^−1^
*T* = 293 K
0.30 × 0.20 × 0.20 mm

### Data collection  

Oxford Diffraction Xcalibur, Sapphire3 diffractometer
Absorption correction: multi-scan (*CrysAlis PRO*; Oxford Diffraction, 2010 ▶) *T*_min_ = 0.850, *T*_max_ = 1.000
10634 measured reflections
4621 independent reflections
2763 reflections with *I* > 2σ(*I*)
*R*_int_ = 0.035

### Refinement  

*R*[*F* ^2^ > 2σ(*F* ^2^)] = 0.051
*wR*(*F* ^2^) = 0.137
*S* = 1.03
4621 reflections
309 parameters
H-atom parameters constrained
Δρ_max_ = 0.27 e Å^−3^
Δρ_min_ = −0.25 e Å^−3^

### 

Data collection: *CrysAlis PRO* (Oxford Diffraction, 2010 ▶); cell refinement: *CrysAlis PRO*; data reduction: *CrysAlis PRO*; program(s) used to solve structure: *SHELXS97* (Sheldrick, 2008 ▶); program(s) used to refine structure: *SHELXL97* (Sheldrick, 2008 ▶); molecular graphics: *ORTEP-3 for Windows* (Farrugia, 2012 ▶) and *PLATON* (Spek, 2009 ▶); software used to prepare material for publication: *PLATON*.

## Supplementary Material

Crystal structure: contains datablock(s) I, Global. DOI: 10.1107/S1600536814017140/su2756sup1.cif

Structure factors: contains datablock(s) I. DOI: 10.1107/S1600536814017140/su2756Isup2.hkl

Supporting information file. DOI: 10.1107/S1600536814017140/su2756Isup3.cml

**Fig. 1.**
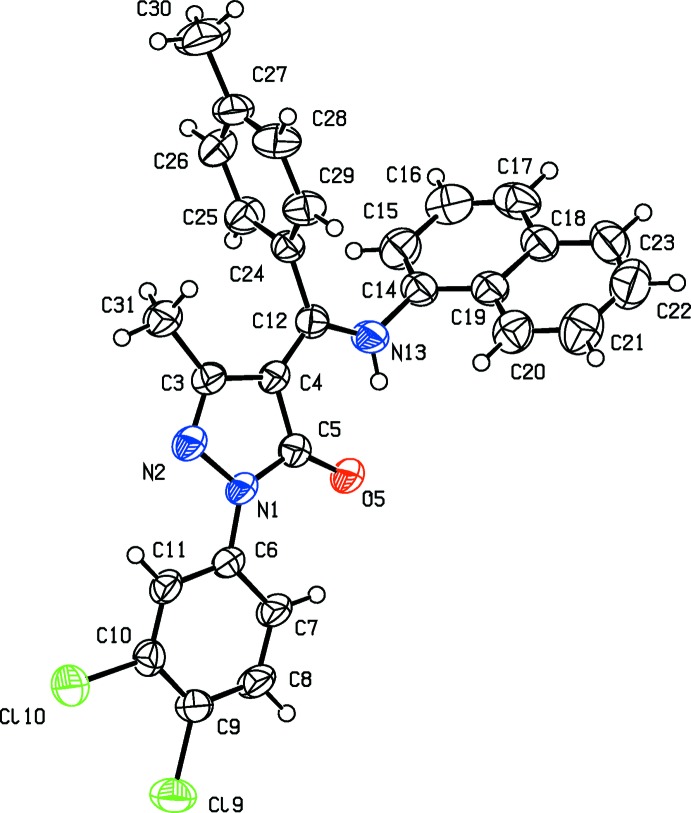
A view of the molecular structure of the title molecule, with atom labelling. The displacement ellipsoids are drawn at the 40% probability level.

**Fig. 2.**
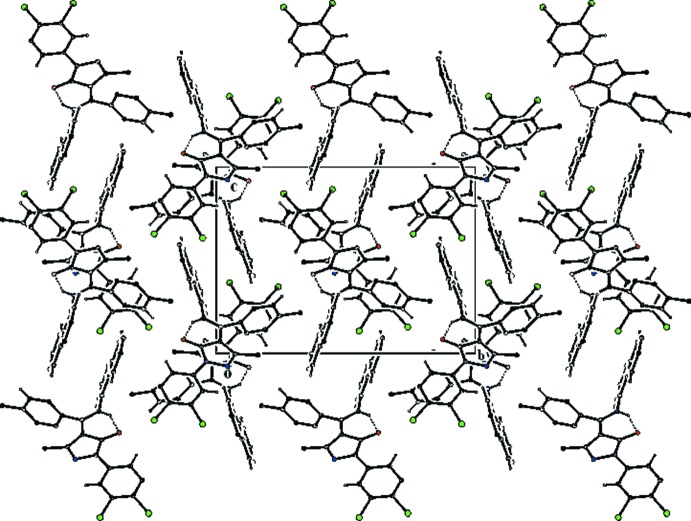
The crystal packing of the title compound viewed along the a axis.

. DOI: 10.1107/S1600536814017140/su2756fig1.tif
A view of the mol­ecular structure of the title mol­ecule, with atom labelling. The displacement ellipsoids are drawn at the 40% probability level.

a . DOI: 10.1107/S1600536814017140/su2756fig2.tif
The crystal packing of the title compound viewed along the *a* axis.

CCDC reference: 1013619

Additional supporting information:  crystallographic information; 3D view; checkCIF report

## Figures and Tables

**Table 1:** Hydrogen-bond geometry (Å, °) *Cg*1, *Cg*3, *Cg*5 are the centroids of rings N1/N2/C3–C5, C14–C19 and C24–C29, respectively.

*D*—H⋯*A*	*D*—H	H⋯*A*	*D*⋯*A*	*D*—H⋯*A*
N13—H13⋯O5	0.86	1.98	2.702 (3)	141
C7—H7⋯O5	0.93	2.32	2.937 (4)	124
C8—H8⋯*Cg*3^i^	0.93	2.71	3.639 (3)	176
C15—H15⋯*Cg*1^ii^	0.93	2.97	3.825 (3)	154
C23—H23⋯*Cg*5^iii^	0.93	2.77	3.679 (3)	165

Symmetry codes: (i) 
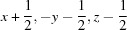
; (ii) 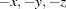; (iii) 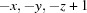.

## supplementary crystallographic information

### S1. Synthesis and crystallization

1-(3,4-Di­chloro­phenyl)-3-methyl-4-(4-methyl­benzoyl)-1*H*-pyrazol-5(4*H*)-one (0.360 g, 1 mmol) was dissolved in a minimum amount of absolute ethanol. To this solution, a solution of 1-amino­naphthalene (0.143 g, 1 mmol) in 10 ml absolute ethanol was added dropwise. The reaction mixture was refluxed for 8 h. After completion of the reaction, it was allowed to cool and then filtered. The filtrate was kept for slow evaporation giving block-like yellow crystals of the title compound in 2–3 days (yield 0.325, 67%). ^1^H NMR (400 MHz,CDCl3, TMS): δ 1.61 (*s*, 3H), 2.48 (*s*, 3H), 6.87 (*d*, 1H), 7.09–7.20 (*m*, 5H), 7.47 (*d*, 1H), 7.55–7.59 (*m*, 1H), 7.62–7.68 (*m*, 2H), 7.85 (*d*, 1H), 8.05–8.08 (*dd*, 1H), 8.22 (*d*, 1H), 8.33 (*d*, 1H), 13.12 (*s*, 1H). Elemental analysis calculated for C_28_H_21_Cl_2_N_3_O: C 69.14, H 4.35, N 8.64; found: C 69.18, H 4.41, 8.65.

### S2. Refinement

Crystal data, data collection and structure refinement details are summarized in Table 2. All the H atoms were fixed geometrically and allowed to ride on their parent atoms: N—H = 0.86 Å, C—H =0.93–0.96 Å with *U*_iso_(H) = 1.5*U*_eq_(C-methyl) and = 1.2*U*_eq_(N,C) for other H atoms.

### Crystal data

**Table:**

C_28_H_21_Cl_2_N_3_O	*F*(000) = 1008
*M**_r_* = 486.38	*D*_x_ = 1.368 Mg m^−^^3^
Monoclinic, *P*2_1_/*n*	Mo *K*α radiation, λ = 0.71073 Å
Hall symbol: -P 2yn	Cell parameters from 2540 reflections
*a* = 11.4621 (8) Å	θ = 3.8–27.7°
*b* = 16.9351 (11) Å	µ = 0.30 mm^−^^1^
*c* = 12.2704 (9) Å	*T* = 293 K
β = 97.478 (6)°	Block, yellow
*V* = 2361.6 (3) Å^3^	0.30 × 0.20 × 0.20 mm
*Z* = 4	

### Data collection

**Table:**

Oxford Diffraction Xcalibur, Sapphire3 diffractometer	4621 independent reflections
Radiation source: fine-focus sealed tube	2763 reflections with *I* > 2σ(*I*)
Graphite monochromator	*R*_int_ = 0.035
Detector resolution: 16.1049 pixels mm^-1^	θ_max_ = 26.0°, θ_min_ = 3.6°
ω scans	*h* = −14→12
Absorption correction: multi-scan (*CrysAlis PRO*; Oxford Diffraction, 2010)	*k* = −20→11
*T*_min_ = 0.850, *T*_max_ = 1.000	*l* = −15→13
10634 measured reflections	

### Refinement

**Table:**

Refinement on *F*^2^	Primary atom site location: structure-invariant direct methods
Least-squares matrix: full	Secondary atom site location: difference Fourier map
*R*[*F*^2^ > 2σ(*F*^2^)] = 0.051	Hydrogen site location: inferred from neighbouring sites
*wR*(*F*^2^) = 0.137	H-atom parameters constrained
*S* = 1.03	*w* = 1/[σ^2^(*F*_o_^2^) + (0.0446*P*)^2^ + 0.2032*P*] where *P* = (*F*_o_^2^ + 2*F*_c_^2^)/3
4621 reflections	(Δ/σ)_max_ < 0.001
309 parameters	Δρ_max_ = 0.27 e Å^−^^3^
0 restraints	Δρ_min_ = −0.25 e Å^−^^3^

### Special details

**Table:**

Geometry. All e.s.d.'s (except the e.s.d. in the dihedral angle between two l.s. planes) are estimated using the full covariance matrix. The cell e.s.d.'s are taken into account individually in the estimation of e.s.d.'s in distances, angles and torsion angles; correlations between e.s.d.'s in cell parameters are only used when they are defined by crystal symmetry. An approximate (isotropic) treatment of cell e.s.d.'s is used for estimating e.s.d.'s involving l.s. planes.
Refinement. Refinement of *F*^2^ against ALL reflections. The weighted *R*-factor *wR* and goodness of fit *S* are based on *F*^2^, conventional *R*-factors *R* are based on *F*, with *F* set to zero for negative *F*^2^. The threshold expression of *F*^2^ > σ(*F*^2^) is used only for calculating *R*-factors(gt) *etc*. and is not relevant to the choice of reflections for refinement. *R*-factors based on *F*^2^ are statistically about twice as large as those based on *F*, and *R*- factors based on ALL data will be even larger.

### Fractional atomic coordinates and isotropic or equivalent isotropic displacement parameters (Å^2^)

**Table:**

	*x*	*y*	*z*	*U*_iso_*/*U*_eq_	
N1	0.64906 (18)	0.03805 (13)	0.04467 (18)	0.0434 (6)	
N2	0.65554 (18)	−0.04357 (14)	0.06786 (19)	0.0482 (6)	
C3	0.7247 (2)	−0.07400 (17)	0.0027 (2)	0.0432 (7)	
C4	0.7673 (2)	−0.01509 (16)	−0.0659 (2)	0.0397 (6)	
C5	0.7151 (2)	0.05828 (17)	−0.0369 (2)	0.0417 (7)	
O5	0.72721 (16)	0.12583 (11)	−0.07388 (16)	0.0528 (5)	
C6	0.5933 (2)	0.08712 (16)	0.1152 (2)	0.0412 (7)	
C7	0.5903 (2)	0.16869 (17)	0.1037 (2)	0.0519 (8)	
H7	0.6216	0.1926	0.0458	0.062*	
C8	0.5406 (2)	0.21407 (18)	0.1789 (2)	0.0545 (8)	
H8	0.5400	0.2687	0.1718	0.065*	
C9	0.4921 (2)	0.17990 (18)	0.2641 (2)	0.0462 (7)	
Cl9	0.43371 (7)	0.23935 (5)	0.35807 (7)	0.0669 (3)	
C10	0.4940 (2)	0.09823 (17)	0.2742 (2)	0.0439 (7)	
Cl10	0.43480 (7)	0.05190 (5)	0.37990 (7)	0.0672 (3)	
C11	0.5437 (2)	0.05215 (17)	0.2003 (2)	0.0433 (7)	
H11	0.5441	−0.0025	0.2075	0.052*	
C12	0.8483 (2)	−0.01823 (17)	−0.1416 (2)	0.0418 (6)	
N13	0.8763 (2)	0.04812 (14)	−0.18956 (18)	0.0537 (6)	
H13	0.8456	0.0905	−0.1673	0.064*	
C14	0.9510 (3)	0.05849 (17)	−0.2735 (2)	0.0489 (7)	
C15	1.0692 (3)	0.04650 (19)	−0.2527 (3)	0.0606 (8)	
H15	1.1023	0.0294	−0.1835	0.073*	
C16	1.1413 (3)	0.0596 (2)	−0.3341 (3)	0.0667 (9)	
H16	1.2219	0.0510	−0.3190	0.080*	
C17	1.0946 (3)	0.0847 (2)	−0.4346 (3)	0.0672 (10)	
H17	1.1434	0.0922	−0.4887	0.081*	
C18	0.9730 (3)	0.09982 (17)	−0.4592 (2)	0.0548 (8)	
C19	0.8994 (2)	0.08688 (16)	−0.3767 (2)	0.0482 (7)	
C20	0.7781 (3)	0.10392 (19)	−0.4008 (3)	0.0616 (9)	
H20	0.7289	0.0967	−0.3470	0.074*	
C21	0.7329 (3)	0.1306 (2)	−0.5011 (3)	0.0781 (11)	
H21	0.6528	0.1414	−0.5154	0.094*	
C22	0.8034 (4)	0.1422 (2)	−0.5832 (3)	0.0845 (11)	
H22	0.7707	0.1603	−0.6520	0.101*	
C23	0.9198 (4)	0.1272 (2)	−0.5626 (3)	0.0750 (10)	
H23	0.9665	0.1352	−0.6182	0.090*	
C24	0.9034 (2)	−0.09451 (16)	−0.1668 (2)	0.0397 (6)	
C25	1.0094 (2)	−0.11772 (19)	−0.1107 (2)	0.0543 (8)	
H25	1.0509	−0.0836	−0.0604	0.065*	
C26	1.0547 (3)	−0.1916 (2)	−0.1288 (3)	0.0583 (8)	
H26	1.1265	−0.2064	−0.0898	0.070*	
C27	0.9968 (3)	−0.24353 (18)	−0.2022 (3)	0.0570 (8)	
C28	0.8905 (3)	−0.21983 (19)	−0.2581 (3)	0.0634 (9)	
H28	0.8487	−0.2542	−0.3079	0.076*	
C29	0.8449 (2)	−0.14606 (18)	−0.2416 (2)	0.0523 (8)	
H29	0.7738	−0.1310	−0.2815	0.063*	
C30	1.0454 (3)	−0.3243 (2)	−0.2191 (4)	0.0959 (13)	
H30A	1.1298	−0.3226	−0.2057	0.144*	
H30B	1.0211	−0.3410	−0.2933	0.144*	
H30C	1.0165	−0.3608	−0.1692	0.144*	
C31	0.7482 (3)	−0.16071 (17)	0.0094 (3)	0.0609 (8)	
H31A	0.7077	−0.1834	0.0656	0.091*	
H31B	0.8312	−0.1696	0.0268	0.091*	
H31C	0.7206	−0.1848	−0.0601	0.091*	

### Atomic displacement parameters (Å^2^)

**Table:**

	*U*^11^	*U*^22^	*U*^33^	*U*^12^	*U*^13^	*U*^23^
N1	0.0540 (13)	0.0289 (13)	0.0494 (14)	0.0057 (11)	0.0149 (11)	0.0070 (11)
N2	0.0589 (14)	0.0312 (13)	0.0564 (15)	0.0011 (12)	0.0142 (12)	0.0053 (12)
C3	0.0488 (15)	0.0346 (16)	0.0461 (16)	0.0037 (14)	0.0059 (13)	−0.0011 (13)
C4	0.0467 (14)	0.0298 (15)	0.0433 (15)	0.0018 (13)	0.0087 (12)	0.0038 (13)
C5	0.0458 (15)	0.0370 (17)	0.0431 (16)	0.0012 (14)	0.0094 (12)	0.0032 (14)
O5	0.0707 (12)	0.0339 (11)	0.0579 (13)	0.0069 (10)	0.0237 (10)	0.0112 (10)
C6	0.0440 (14)	0.0346 (16)	0.0461 (16)	0.0079 (13)	0.0102 (12)	0.0039 (13)
C7	0.0650 (18)	0.0315 (16)	0.0640 (19)	0.0078 (15)	0.0269 (15)	0.0102 (15)
C8	0.0636 (18)	0.0302 (16)	0.074 (2)	0.0087 (15)	0.0252 (16)	0.0090 (15)
C9	0.0473 (15)	0.0417 (18)	0.0510 (17)	0.0027 (14)	0.0117 (13)	−0.0034 (14)
Cl9	0.0817 (5)	0.0506 (5)	0.0738 (6)	0.0022 (4)	0.0307 (4)	−0.0125 (4)
C10	0.0454 (15)	0.0421 (17)	0.0448 (16)	−0.0018 (14)	0.0078 (12)	0.0076 (14)
Cl10	0.0858 (6)	0.0564 (6)	0.0659 (5)	0.0014 (5)	0.0339 (4)	0.0135 (4)
C11	0.0482 (14)	0.0316 (16)	0.0509 (17)	0.0035 (13)	0.0089 (13)	0.0079 (13)
C12	0.0508 (15)	0.0354 (16)	0.0383 (15)	−0.0001 (14)	0.0023 (12)	−0.0024 (13)
N13	0.0771 (16)	0.0327 (14)	0.0571 (15)	0.0004 (13)	0.0310 (13)	0.0000 (12)
C14	0.0653 (18)	0.0353 (17)	0.0491 (18)	−0.0056 (15)	0.0181 (14)	−0.0042 (14)
C15	0.0592 (19)	0.054 (2)	0.068 (2)	−0.0017 (17)	0.0068 (16)	0.0021 (17)
C16	0.0575 (19)	0.055 (2)	0.090 (3)	−0.0042 (17)	0.0204 (19)	−0.005 (2)
C17	0.077 (2)	0.053 (2)	0.080 (3)	−0.0107 (18)	0.042 (2)	−0.009 (2)
C18	0.083 (2)	0.0346 (17)	0.0509 (19)	−0.0100 (16)	0.0230 (17)	−0.0052 (15)
C19	0.0623 (18)	0.0332 (16)	0.0510 (18)	−0.0070 (14)	0.0149 (14)	−0.0057 (14)
C20	0.063 (2)	0.053 (2)	0.069 (2)	−0.0078 (17)	0.0115 (17)	0.0049 (18)
C21	0.079 (2)	0.070 (3)	0.080 (3)	−0.004 (2)	−0.009 (2)	0.005 (2)
C22	0.115 (3)	0.064 (3)	0.071 (3)	0.003 (2)	−0.001 (2)	0.008 (2)
C23	0.121 (3)	0.056 (2)	0.052 (2)	−0.008 (2)	0.026 (2)	−0.0032 (18)
C24	0.0468 (15)	0.0332 (15)	0.0403 (15)	−0.0027 (13)	0.0106 (12)	−0.0022 (13)
C25	0.0587 (17)	0.0477 (19)	0.0533 (18)	0.0043 (16)	−0.0047 (14)	−0.0079 (16)
C26	0.0609 (18)	0.049 (2)	0.065 (2)	0.0150 (17)	0.0069 (15)	0.0080 (17)
C27	0.071 (2)	0.0380 (18)	0.068 (2)	0.0078 (17)	0.0304 (17)	−0.0028 (16)
C28	0.071 (2)	0.048 (2)	0.072 (2)	−0.0026 (17)	0.0125 (17)	−0.0232 (18)
C29	0.0501 (16)	0.0466 (19)	0.0591 (19)	−0.0009 (15)	0.0034 (14)	−0.0142 (16)
C30	0.120 (3)	0.048 (2)	0.126 (3)	0.023 (2)	0.039 (3)	−0.010 (2)
C31	0.082 (2)	0.0364 (18)	0.069 (2)	0.0046 (16)	0.0275 (17)	0.0054 (16)

### Geometric parameters (Å, º)

**Table:**

N1—C5	1.375 (3)	C17—C18	1.411 (4)
N1—N2	1.411 (3)	C17—H17	0.9300
N1—C6	1.412 (3)	C18—C23	1.413 (4)
N2—C3	1.303 (3)	C18—C19	1.416 (4)
C3—C4	1.431 (3)	C19—C20	1.414 (4)
C3—C31	1.493 (4)	C20—C21	1.350 (4)
C4—C12	1.397 (3)	C20—H20	0.9300
C4—C5	1.443 (4)	C21—C22	1.385 (5)
C5—O5	1.245 (3)	C21—H21	0.9300
C6—C11	1.385 (3)	C22—C23	1.350 (5)
C6—C7	1.389 (4)	C22—H22	0.9300
C7—C8	1.379 (4)	C23—H23	0.9300
C7—H7	0.9300	C24—C25	1.374 (4)
C8—C9	1.374 (4)	C24—C29	1.376 (3)
C8—H8	0.9300	C25—C26	1.384 (4)
C9—C10	1.389 (4)	C25—H25	0.9300
C9—Cl9	1.729 (3)	C26—C27	1.367 (4)
C10—C11	1.374 (3)	C26—H26	0.9300
C10—Cl10	1.729 (3)	C27—C28	1.378 (4)
C11—H11	0.9300	C27—C30	1.501 (4)
C12—N13	1.327 (3)	C28—C29	1.379 (4)
C12—C24	1.488 (4)	C28—H28	0.9300
N13—C14	1.432 (3)	C29—H29	0.9300
N13—H13	0.8600	C30—H30A	0.9600
C14—C15	1.362 (4)	C30—H30B	0.9600
C14—C19	1.410 (4)	C30—H30C	0.9600
C15—C16	1.394 (4)	C31—H31A	0.9600
C15—H15	0.9300	C31—H31B	0.9600
C16—C17	1.348 (4)	C31—H31C	0.9600
C16—H16	0.9300		
			
C5—N1—N2	111.8 (2)	C17—C18—C23	123.4 (3)
C5—N1—C6	129.5 (2)	C17—C18—C19	118.9 (3)
N2—N1—C6	118.0 (2)	C23—C18—C19	117.7 (3)
C3—N2—N1	106.5 (2)	C14—C19—C20	122.9 (3)
N2—C3—C4	111.5 (2)	C14—C19—C18	118.4 (3)
N2—C3—C31	118.3 (2)	C20—C19—C18	118.7 (3)
C4—C3—C31	130.2 (2)	C21—C20—C19	120.6 (3)
C12—C4—C3	132.2 (3)	C21—C20—H20	119.7
C12—C4—C5	121.9 (2)	C19—C20—H20	119.7
C3—C4—C5	105.8 (2)	C20—C21—C22	121.3 (3)
O5—C5—N1	126.3 (2)	C20—C21—H21	119.3
O5—C5—C4	129.2 (2)	C22—C21—H21	119.3
N1—C5—C4	104.5 (2)	C23—C22—C21	119.6 (4)
C11—C6—C7	119.7 (2)	C23—C22—H22	120.2
C11—C6—N1	118.3 (2)	C21—C22—H22	120.2
C7—C6—N1	122.0 (2)	C22—C23—C18	122.0 (3)
C8—C7—C6	119.6 (3)	C22—C23—H23	119.0
C8—C7—H7	120.2	C18—C23—H23	119.0
C6—C7—H7	120.2	C25—C24—C29	118.4 (3)
C9—C8—C7	121.2 (3)	C25—C24—C12	121.0 (2)
C9—C8—H8	119.4	C29—C24—C12	120.5 (2)
C7—C8—H8	119.4	C24—C25—C26	120.3 (3)
C8—C9—C10	118.9 (3)	C24—C25—H25	119.9
C8—C9—Cl9	119.5 (2)	C26—C25—H25	119.9
C10—C9—Cl9	121.6 (2)	C27—C26—C25	121.8 (3)
C11—C10—C9	120.7 (2)	C27—C26—H26	119.1
C11—C10—Cl10	118.3 (2)	C25—C26—H26	119.1
C9—C10—Cl10	121.0 (2)	C26—C27—C28	117.6 (3)
C10—C11—C6	120.0 (3)	C26—C27—C30	121.3 (3)
C10—C11—H11	120.0	C28—C27—C30	121.1 (3)
C6—C11—H11	120.0	C27—C28—C29	121.2 (3)
N13—C12—C4	118.9 (2)	C27—C28—H28	119.4
N13—C12—C24	120.6 (2)	C29—C28—H28	119.4
C4—C12—C24	120.5 (2)	C24—C29—C28	120.8 (3)
C12—N13—C14	128.6 (2)	C24—C29—H29	119.6
C12—N13—H13	115.7	C28—C29—H29	119.6
C14—N13—H13	115.7	C27—C30—H30A	109.5
C15—C14—C19	120.6 (3)	C27—C30—H30B	109.5
C15—C14—N13	121.4 (3)	H30A—C30—H30B	109.5
C19—C14—N13	117.9 (2)	C27—C30—H30C	109.5
C14—C15—C16	120.7 (3)	H30A—C30—H30C	109.5
C14—C15—H15	119.7	H30B—C30—H30C	109.5
C16—C15—H15	119.7	C3—C31—H31A	109.5
C17—C16—C15	120.3 (3)	C3—C31—H31B	109.5
C17—C16—H16	119.9	H31A—C31—H31B	109.5
C15—C16—H16	119.9	C3—C31—H31C	109.5
C16—C17—C18	121.1 (3)	H31A—C31—H31C	109.5
C16—C17—H17	119.5	H31B—C31—H31C	109.5
C18—C17—H17	119.5		
			
C5—N1—N2—C3	0.4 (3)	C24—C12—N13—C14	4.8 (4)
C6—N1—N2—C3	−170.9 (2)	C12—N13—C14—C15	−68.8 (4)
N1—N2—C3—C4	0.1 (3)	C12—N13—C14—C19	115.5 (3)
N1—N2—C3—C31	179.9 (2)	C19—C14—C15—C16	−2.1 (5)
N2—C3—C4—C12	174.9 (3)	N13—C14—C15—C16	−177.7 (3)
C31—C3—C4—C12	−4.9 (5)	C14—C15—C16—C17	0.3 (5)
N2—C3—C4—C5	−0.5 (3)	C15—C16—C17—C18	1.3 (5)
C31—C3—C4—C5	179.7 (3)	C16—C17—C18—C23	179.1 (3)
N2—N1—C5—O5	−179.2 (3)	C16—C17—C18—C19	−1.1 (5)
C6—N1—C5—O5	−9.3 (4)	C15—C14—C19—C20	−176.9 (3)
N2—N1—C5—C4	−0.6 (3)	N13—C14—C19—C20	−1.2 (4)
C6—N1—C5—C4	169.3 (2)	C15—C14—C19—C18	2.2 (4)
C12—C4—C5—O5	3.2 (5)	N13—C14—C19—C18	178.0 (2)
C3—C4—C5—O5	179.2 (3)	C17—C18—C19—C14	−0.7 (4)
C12—C4—C5—N1	−175.3 (2)	C23—C18—C19—C14	179.1 (3)
C3—C4—C5—N1	0.7 (3)	C17—C18—C19—C20	178.5 (3)
C5—N1—C6—C11	−172.1 (2)	C23—C18—C19—C20	−1.7 (4)
N2—N1—C6—C11	−2.7 (3)	C14—C19—C20—C21	−179.6 (3)
C5—N1—C6—C7	5.8 (4)	C18—C19—C20—C21	1.2 (5)
N2—N1—C6—C7	175.2 (2)	C19—C20—C21—C22	−0.1 (5)
C11—C6—C7—C8	1.6 (4)	C20—C21—C22—C23	−0.5 (6)
N1—C6—C7—C8	−176.2 (2)	C21—C22—C23—C18	0.0 (6)
C6—C7—C8—C9	−1.1 (4)	C17—C18—C23—C22	−179.1 (3)
C7—C8—C9—C10	0.3 (4)	C19—C18—C23—C22	1.1 (5)
C7—C8—C9—Cl9	178.6 (2)	N13—C12—C24—C25	86.4 (3)
C8—C9—C10—C11	0.1 (4)	C4—C12—C24—C25	−92.9 (3)
Cl9—C9—C10—C11	−178.18 (19)	N13—C12—C24—C29	−98.4 (3)
C8—C9—C10—Cl10	179.7 (2)	C4—C12—C24—C29	82.4 (3)
Cl9—C9—C10—Cl10	1.5 (3)	C29—C24—C25—C26	−0.7 (4)
C9—C10—C11—C6	0.4 (4)	C12—C24—C25—C26	174.6 (3)
Cl10—C10—C11—C6	−179.25 (19)	C24—C25—C26—C27	0.2 (5)
C7—C6—C11—C10	−1.3 (4)	C25—C26—C27—C28	−0.3 (4)
N1—C6—C11—C10	176.6 (2)	C25—C26—C27—C30	−178.5 (3)
C3—C4—C12—N13	−176.0 (3)	C26—C27—C28—C29	0.8 (5)
C5—C4—C12—N13	−1.2 (4)	C30—C27—C28—C29	179.1 (3)
C3—C4—C12—C24	3.2 (4)	C25—C24—C29—C28	1.3 (4)
C5—C4—C12—C24	178.0 (2)	C12—C24—C29—C28	−174.1 (3)
C4—C12—N13—C14	−176.0 (2)	C27—C28—C29—C24	−1.4 (5)

### Hydrogen-bond geometry (Å, º)

Cg1, Cg3, Cg5 are the centroids of rings N1/N2/C3–C5, C14–C19 and C24–C29, respectively.

**Table:**

*D*—H···*A*	*D*—H	H···*A*	*D*···*A*	*D*—H···*A*
N13—H13···O5	0.86	1.98	2.702 (3)	141
C7—H7···O5	0.93	2.32	2.937 (4)	124
C8—H8···*Cg*3^i^	0.93	2.71	3.639 (3)	176
C15—H15···*Cg*1^ii^	0.93	2.97	3.825 (3)	154
C23—H23···*Cg*5^iii^	0.93	2.77	3.679 (3)	165

Symmetry codes: (i) *x*+1/2, −*y*−1/2, *z*−1/2; (ii) −*x*, −*y*, −*z*; (iii) −*x*, −*y*, −*z*+1.
