# Supplementary figures and images for: Crystal structure of (Z)-1-(3,4-dichlorophenyl)-3-methyl-4-[(naphthalen-1-yl­amino)(p-tolyl)methylidene]-1H-pyrazol-5(4H)-one
Source: Acta Crystallogr Sect E Struct Rep Online. 2014 Aug 1;70(Pt 9):o955–6. doi: 10.1107/S1600536814017140 (PMC4186144; doi:10.1107/S1600536814017140)

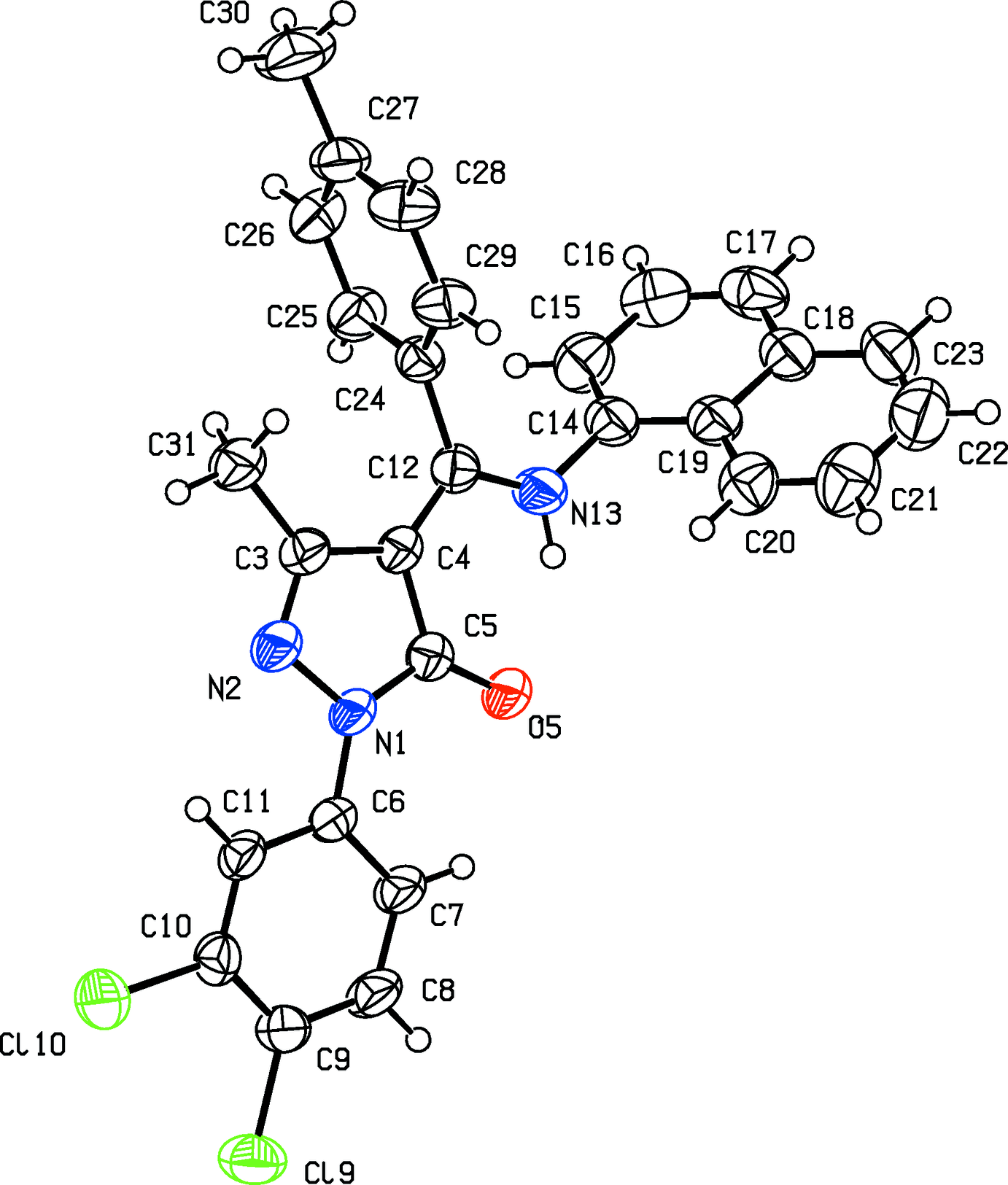

Supplement: Supplementary file 4 [file e-70-0o955-fig1.tif]

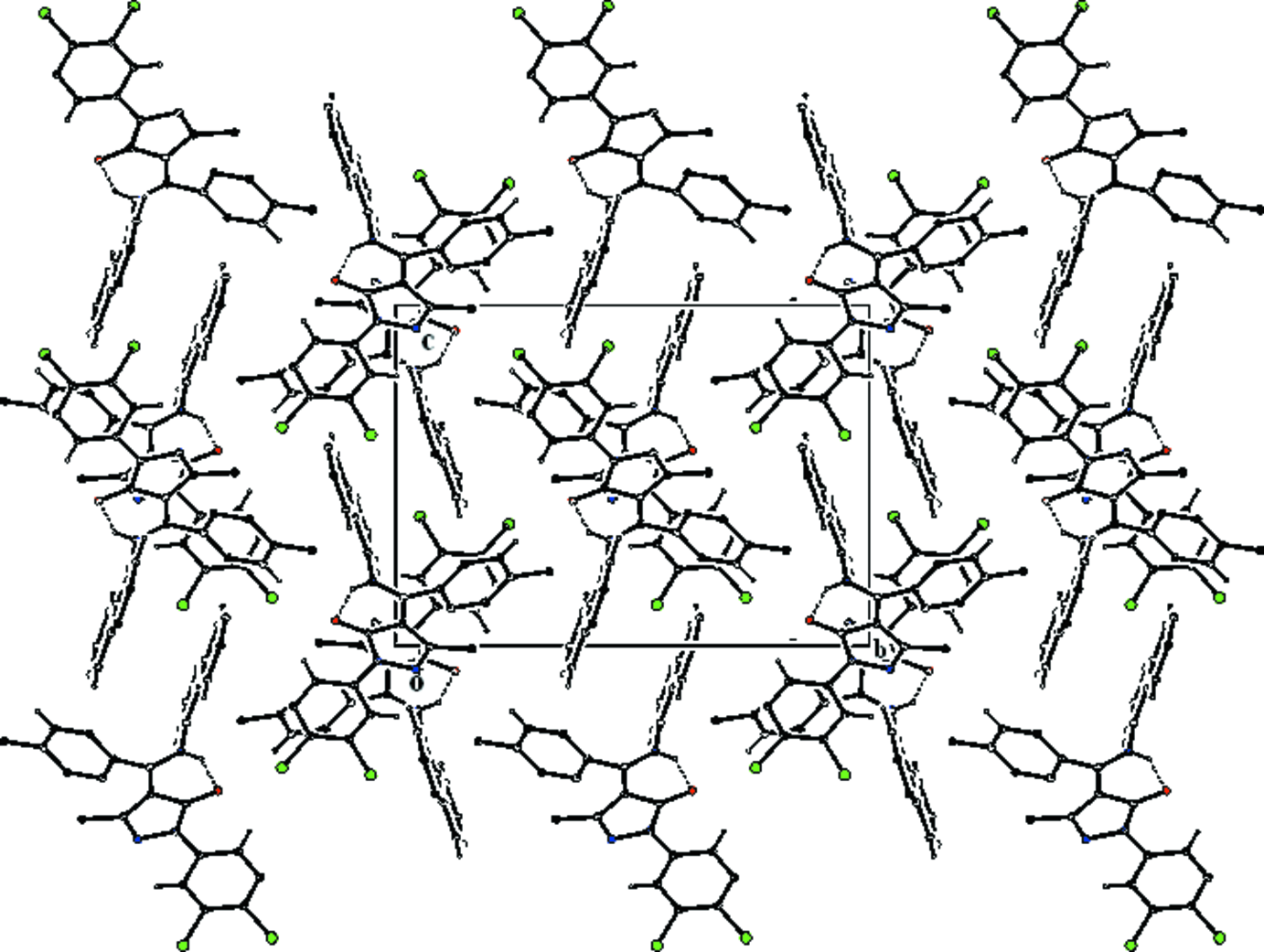

Supplement: Supplementary file 5 [file e-70-0o955-fig2.tif]
